# Human-Robot Collaboration for Healthcare: A Narrative Review

**DOI:** 10.7759/cureus.49210

**Published:** 2023-11-21

**Authors:** Induni N Weerarathna, David Raymond, Anurag Luharia

**Affiliations:** 1 Biomedical Sciences, School of Allied Health Sciences, Datta Meghe Institute of Higher Education and Research, Wardha, IND; 2 Computer Science and Medical Engineering, Datta Meghe Institute of Higher Education and Research, Wardha, IND; 3 Radiotherapy, Jawaharlal Nehru Medical College, Datta Meghe Institute of Higher Education and Research, Wardha, IND

**Keywords:** challenges, ethical considerations, robotic assistance surgery, telemedicine, robots

## Abstract

Robotic applications have often quickly transitioned from industrial to social. Because of this, robots can now engage with people in a natural way and blend in with their surroundings. Due to the lack of medical professionals, growing healthcare costs, and the exponential rise in the population of vulnerable groups like the ill, elderly, and children with developmental disabilities, the use of social robots in the healthcare system is expanding. As a result, social robots are employed in the medical field to entertain and educate hospitalized patients about health issues, as well as to assist the elderly and sick. They are also employed in the dispensing of medications, rehabilitation, and emotional and geriatric care. Thus, social robots raise the standard and effectiveness of medical care. This article explains how patients and healthcare professionals collaborate with robots in the healthcare industry. The objectives of this collaboration are to resolve moral and legal concerns, improve patient outcomes, and improve healthcare delivery. It has a broad range of uses, including telemedicine, rehabilitation, and robotic surgical support. Human-robot interaction is the term used to describe interactions between social robots and people. Many obstacles stand in the way of human-robot interaction in healthcare, including safety concerns, acceptability issues, appropriateness, usefulness, and the worry that robots may replace human carers. In the end, these difficulties result in a poor adoption rate for robotic technology. As a result, the applications and difficulties of human-robot interaction in healthcare are thoroughly evaluated in this research. This study also reviews future safety prospects from human-robot interaction in healthcare, as well as ethical and usability issues including privacy, trust, and safety, and our aims to provide a comprehensive overview of the use of robots in healthcare, including their applications, benefits, challenges, and prospects, to facilitate a deeper understanding of this evolving field.

## Introduction and background

Often called a cobot, a collaborative robot is a kind of robot made to operate side by side with people in a shared workspace, encouraging collaboration and communication between people and machines. Collaborative robots are built with sophisticated sensors and safety mechanisms to allow them to work near people without endangering them, in contrast to standard industrial robots, which are usually housed in cages for security reasons. These robots recognize and adjust to the presence of humans using a variety of technologies, including force and torque sensors, vision systems, and machine learning algorithms, assuring a productive and safe working environment. Although telecommunications-related computers and cameras help to facilitate the remote monitoring and management of cobots, the core of collaborative robotics is human-machine contact and cooperation in a shared workspace [1]. Imagine living in a society where people and robots often interact and live, akin to the 1960s animated series The Jetsons, which was produced by Hanna-Barbera. Even while flying cars and skyscraper mansions are still a ways off, as technology advances, life as we know it is gradually beginning to resemble the program. Robotics is being used more for social contact, including in healthcare services, than for industrial purposes. Due to its ability to save time and improve accuracy, assistive technology is becoming increasingly important in the healthcare sector [2].

There is currently a scarcity of qualified workers in the medical field. Because of the ageing population, there is an increasing need for healthcare personnel, which has resulted in a shortage of trained individuals in the medical field. This difficulty is highlighted by recent data, which show a significant scarcity of medical specialists in a range of specialisations. The need for healthcare workers is projected to increase as the demographic landscape changes and the ageing population becomes an increasingly large portion of the population. This confluence of dwindling labour supply and rising demand draws attention to a crucial social and economic problem and underscores the need for focused measures to close the widening skills gap in the medical field. Social and economic concern is the increased need for healthcare personnel brought on by the aging population. Shortly, it is anticipated that human-robot collaboration in healthcare will increase since it might be very beneficial to relieve medical personnel of their technical systems. Involving the medical team from the outset is crucial to promoting acceptance of these technological systems and digital assistance. Thus, this study set out to investigate disparities in the general perception and acceptability of robots in the healthcare industry [3].

In medical contexts, collaborative robots, or cobots, are being used to help doctors with rehabilitation, patients with mental health issues, and people with impairments [4]. Cobots would lessen healthcare personnel burnout due to the significant rise in patients in hospitals and the exponential rise in demand for telerehabilitation. With the advancement of technology and the impact of pandemics like coronavirus disease 2019 (COVID-19) on people's lives, greater faith should be placed in human-robot interaction in healthcare services. The desire for little human contact has changed the way in-person healthcare is offered, making telehealth more crucial than ever. Robots used in telehealth enable doctor-patient communication without posing a risk to safety. To diagnose COVID-19, for instance, there are diagnostic robots that can consult with patients, take their temperatures, identify coughs, and more [5]. Telehealth robots help healthcare professionals and patients by facilitating communication between patients and their loved ones. These robots can record data, monitor patient status, and alert staff in an emergency [6]. Robots are supposed to collaborate with doctors and/or patients in each of the aforementioned scenarios to provide the best possible result. They can take the role of therapists in certain situations. Since patients must be trusted, human-robot interaction is a topic that is gradually being introduced into the medical community. The research was done through questionnaires and experiments to determine what qualities a robot should have to foster more trust between humans and robots in healthcare settings, especially physical therapy.

The majority of assistive robots are still prototypes that can only vaguely mimic the dynamics of human-robot interaction. In record time, researchers are trying to construct a being that can help us, inspire us, teach us, and support heavy lifting and precision work, perhaps becoming the ideal interactive companion depending on its intended application [7]. But to achieve this, significant advancements must be made beyond the current state-of-the-art robotic implementations, which rely heavily on nonverbal communication as the foundation for human-robot interaction (HRI). The analysis of the HRI is still a relatively young field of study. The area of study is wide and varied, and the design processes for both hardware and software face intriguing and unresolved issues. The scientific community is now investigating the usage of assistive robots in a variety of sectors. For these reasons, a variety of disciplines-from those with a more mathematical engineering bent to the more humanistic sciences-have contributed to the development of the HRI. After that, the theme is expanded to include a wide range of still-being-explored ethical and legal concerns [8].

Robots that collaborate can be useful. One of the most promising fields for process and business innovation is collaborative robots. Specifically, in the medical field, there are several monotonous, low-value jobs that healthcare professionals perform that could be assigned to collaborative robots [9]. Thus, it is essential that these robots be simple to operate and allow users to specify tasks for them. Increasingly, collaborative robots are being used in a variety of industries to do activities that can profit from the fusion of robotic efficiency and precision with human intuition and dexterity. Cobots are used in manufacturing to assist human workers in tasks including pick-and-place operations, assembly, and quality control. This increases the overall production efficiency. Collaborative robots are used in warehousing and logistics for forklift operations as well as packing and sorting commodities. Cobots in the medical field can handle materials in sterile settings or help with repetitive chores in labs. Collaborative robots are also becoming more common in industries like agriculture, where they are used for activities like harvesting and sorting food. The fact that these applications are still being investigated shows how cobots can improve human capabilities, expedite procedures, and create safer, more efficient work environments [10].

All things considered, the subject of robotics in pharmacy is expanding quickly, and technology is always advancing. Robots have the potential to completely transform pharmacy practice as they grow more complex and advanced, enhancing patient safety and boosting productivity [11]. The pharmaceutical business has seen a rise in the use of cobots because of their adaptability and tight collaboration with human operators. In the pharmaceutical business, cobots' capacity to supplement human labor and increase industrial processes' productivity and efficiency is one of its main advantages. Pharmaceutical firms can enhance production and output while upholding stringent product quality standards by incorporating robots into their operations. Cobots can also improve worker safety by performing hazardous and repetitive jobs that could endanger human workers. In the pharmaceutical business, cobots are commonly used for a variety of functions, such as quality monitoring, assembly, and packaging. Cobots are versatile and can be readily programmed to do a variety of activities, which enables them to work with a range of products and on several manufacturing lines. Cobots can also be equipped with sensors and cameras, which allows them to do quality checks and exact measurements while in production [12].

Convergent research fields in physical human-robot partnerships, such as sensing, manipulation, and autonomy, will enable highly versatile and adaptable medical robots with improved feel, touch, and decision-making abilities to address clinical issues in the acute treatment of infectious diseases. Robotics can improve patient care and provider safety in three main ways: by reducing carer contact with infected patients to reduce the spread of infection; by enhancing clinical providers' capacity and efficiency to free them up to focus on critical tasks; and by limiting the number of times providers enter an isolation area, which reduces the need for personal protective equipment (PPE) supplies. To address these problems, the state of the art in medical robotics is summed up in this comment [13]. It makes suggestions for utilizing cutting-edge technologies to facilitate the creation of extremely adaptable and versatile robots in case of an outbreak of infectious diseases in the future. All things considered, to increase robots' senses of feeling, touch, and decision-making, breakthroughs in autonomy, manipulation, and sensing are needed [14].

This review article is dedicated to exploring the evolving landscape of human-robot collaborations for healthcare. Our primary aim is to provide a comprehensive and insightful overview of this dynamic field, offering a deep understanding of the current state, challenges, opportunities, and prospects in the intersection of human healthcare and robotic technologies. We will examine a diverse array of robots, ranging from assistive and surgical robots to telemedicine and autonomous healthcare systems, with a focus on their collaborative roles in healthcare delivery. Our scope encompasses various facets of this collaboration, including applications in rehabilitation, elderly care, surgery assistance, and patient monitoring. We will delve into the critical realm of human-robot interaction, examining interfaces, communication modalities, and trust dynamics. Additionally, we will assess the benefits and challenges of integrating robots into healthcare settings, address regulatory and ethical considerations, and provide insights into ongoing research and development efforts. By doing so, this review seeks to enlighten researchers, healthcare professionals, policymakers, and the general public about the transformative potential of human-robot collaboration in healthcare, shaping the future of healthcare delivery and enhancing patient outcomes. Understanding the dynamics of human-robot collaborations for healthcare holds immense significance for both healthcare professionals and patients alike. For healthcare professionals, it offers the potential to enhance the quality and efficiency of care delivery.

Robotic assistance can alleviate physical burdens, enable precision in medical procedures, and provide continuous patient monitoring. These innovations empower healthcare providers to focus more on critical decision-making and personalized patient interactions. For patients, this means improved healthcare access, reduced hospitalization times, and better treatment outcomes. Understanding this topic allows patients to embrace the idea of collaborative care where robots and healthcare professionals work together to ensure their well-being. It's not just a technological advancement; it's a paradigm shift that promises more accessible, efficient, and patient-centered healthcare. This review article aims to provide a comprehensive overview of the use of robots in healthcare, including their applications, benefits, challenges, and prospects, to facilitate a deeper understanding of this evolving field.

## Review

Search methodology

In pursuit of relevant content for this comprehensive review article on the dynamic field of human robots collaboration for health care, an exhaustive search strategy was meticulously executed. The primary aim was to offer a comprehensive overview of the latest advancements, methodologies, and collaborative efforts within various domains of healthcare involving both humans and robots. A systematic exploration was conducted across reputable scholarly databases, including PubMed, Web of Science, Scopus, and Google Scholar, encompassing studies from 2004 to the present to encompass the most recent research findings. To ensure the inclusivity of pertinent studies, these databases were queried with precise and tailored keywords relevant to the subject matter. Keywords such as 'Robots assistance surgery,' 'Telemedicine,' 'Social robots,' 'Ethical considerations,' and 'Treatment' were strategically employed. This meticulous keyword strategy was instrumental in the holistic retrieval of studies that intersected at the junction of human-robot collaboration in healthcare, ensuring a comprehensive and up-to-date analysis.

**Figure 1:**
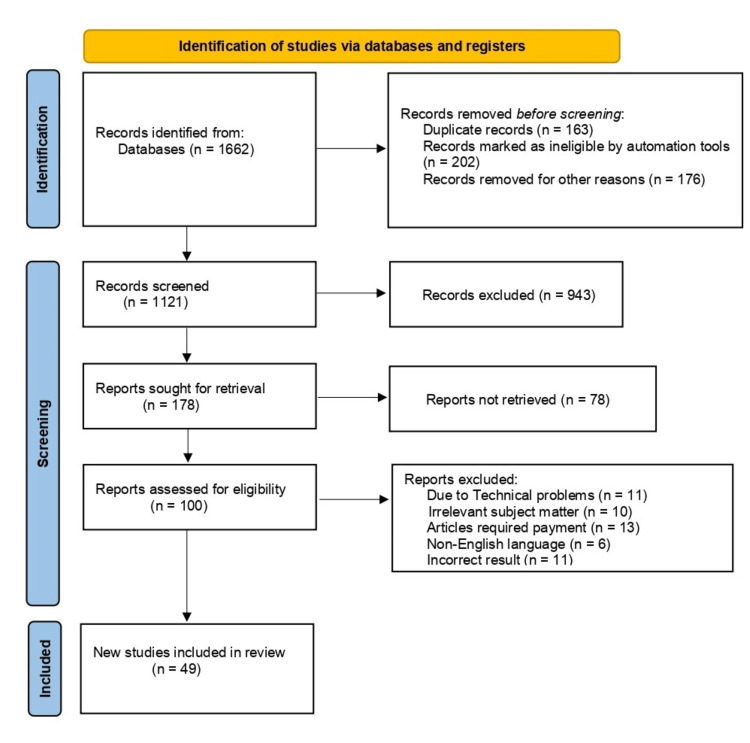
PRISMA flow diagram for literature search Adapted from the Preferred Reporting Items for Systemic Reviews and Meta-analyses (PRISMA) guidelines

Articles, reviews, and reports were carefully curated using predefined inclusion and exclusion criteria specifically tailored to the field of human-robot collaboration for healthcare. Inclusion criteria required that the selected sources be available in the English language and address recent developments in the integration of robotics within healthcare settings. This encompassed a broad spectrum of applications, from surgical robotics to telemedicine and rehabilitation. The review focused on studies conducted between 2004 to 2023, ensuring that the analysis was based on the latest and most relevant research in the field. This rigorous selection process was designed to amass a current and comprehensive body of literature, offering insights into the multifaceted dynamics of human-robot collaboration in healthcare. The data extracted from these chosen studies were meticulously examined and synthesized to provide a nuanced understanding of the role of robots in advancing healthcare practices and the potential implications for future clinical applications (Figure 1).

Results

Throughout the comprehensive literature study on human-robot collaborations for healthcare, several noteworthy conclusions and recurrent themes surfaced. The studied literature continuously emphasizes how collaborative robots can improve healthcare outcomes by helping with surgeries and offering individualized patient care. The use of artificial intelligence in healthcare robotics, the increased emphasis on human-robot interaction design, and the moral dilemmas including patient privacy and permission are some noteworthy trends. Important studies in this field have addressed regulatory issues and highlighted the significance of smooth collaboration between robots and healthcare practitioners. Together, these results show an area full of innovation and the potential to completely transform healthcare delivery for the good of both patients and healthcare professionals. The below subtopics should explore a different aspect of the collaboration.

Robotic Assistance Surgery and Medical Procedures

A surgical robot is a self-powered, computer-controlled apparatus that can be trained to assist the surgeon in doing more difficult tasks by helping to position and manipulate surgical tools [15]. Robotic surgery is one of the most promising applications of HRI in healthcare. It has been demonstrated that robotic surgical systems, like the da Vinci Surgical System, improve patient outcomes by giving surgeons more control, dexterity, and precision [16]. These systems make minimally invasive surgeries possible, which can help patients heal more quickly from injuries, lose less blood, and spend less time in the hospital. Furthermore, robotic surgical devices can lessen the possibility of human error and surgeon fatigue, which will enhance patient outcomes [17].

Surgical robots are utilized to facilitate in-person and remote surgical procedures that lead to less invasive procedures, better precision, enhanced operator eyesight, and exposure protection for both the patient and medical staff [2]. Any kind of surgery carried out with the use of robotic equipment is referred to as robotic surgery or robot-assisted surgery. To improve the skills of surgeons conducting open surgery and get beyond the drawbacks of previous minimally invasive surgical techniques, robotically assisted surgery was created [16]. When performing dissection, hemostasis, and resection during robotically assisted minimally invasive surgery, the surgeon can choose to use a direct telemanipulator or computer control, as an alternative to directly manipulating the tools. Two cutting-edge uses of robots connected to telecommunication networks, like SOCRATES (Computer Motion), are telepresence surgery and robotic telementoring. When doing telerobotic surgery, the surgeon works from the console thousands of kilometers away from the patient-mounted robotic arm. Fiber-optic cables are used to transmit the surgeon's commands to the slave manipulator [18].

Robotic aid in medicine, especially during surgery, is a revolutionary development in the field of medicine. These robotic devices enhance surgeons' abilities and improve patient outcomes by operating in multiple medical specialties. They are not limited to any one area [19]. To fully utilize the potential of new technologies, healthcare personnel must undergo extensive training and skill development. Furthermore, a lot of healthcare facilities may find it expensive to purchase and operate robotic surgical equipment, which emphasizes the necessity of improving accessibility measures [20]. At the heart of these operations is human-robot collaboration, wherein surgeons collaborate with robotic devices to get optimal precision and patient safety. The combination of augmented reality, haptic feedback, and artificial intelligence (AI)-driven decision support is expanding the realm of what is possible as technology advances [21]. This technological revolution is accompanied by ethical and legal considerations, like as liability and patient permission. Prospects for robotic help in healthcare are bright, with the possibility of even more flexible and adaptable devices as well as a wider range of uses in routine medical practice [22]. Recent research on the effects of robotic aid in surgery has shown promise for improving patient outcomes and operating room productivity in several areas. Robotic-assisted operations, especially with systems like the da Vinci Surgical System, have demonstrated promise in improving patient outcomes, albeit outcomes can vary depending on the type of surgery and the surgeon's skill. According to certain research, individuals undergoing robotically assisted treatments might require fewer incisions, less blood loss, and shorter hospital stays than those undergoing traditional surgery. To achieve these results, robotic systems may contribute with their precision and maneuverability. It is imperative to acknowledge that the efficacy of robotic aid is contingent upon various aspects, including the surgeon's level of expertise and the intricacy of the surgical procedure [23].

Robotic Telemedicine and Its Role in Remote Healthcare

The term used for the cutting-edge medical technology that enables communication between patients and doctors even when there is no physical contact is telemedicine. When this technology was first discussed, it was thought to offer a chance for residents in rural and isolated places to get safe, excellent, and necessary healthcare services [24]. Numerous applications of robots in the healthcare system exist. Robots in healthcare are employed for telemedicine, social contact, surgical support, rehabilitation, company, and amusement [25]. Social robots are intended to offer personalized, low-cost, home-based telemedicine technology for both curative and preventive care. Theories that are based on self-reported qualities are used to characterize these robots in terms of inference [26]. Telepresence robots in the healthcare industry allow medical practitioners to be in a remote place while delivering patient care [27]. Telehealth robots can be used to provide patient monitoring and management such as daily vital measurements [28]. Robots assist healthcare workers in their everyday activities in addition to providing direct patient care, which helps to increase productivity and lower the possibility of mistakes. Robots, for instance, can be utilized to move supplies, food, and medication throughout a hospital, freeing up employees to concentrate on other important duties. Robots are now being used by some hospitals to aid with duties like cleaning patient rooms, which lowers the possibility of hospital-acquired diseases [29]. Particularly in the clinical field, there is a notable need for robots as a result of the rising number of COVID-19 patients. Since contaminated surfaces and close human contact are the main ways that SARS-CoV-2 spreads, keeping social distance has become essential as a protective approach. This necessitates treating patients with the least amount of doctor-patient contact possible. Robots can partially replace certain medical jobs, therefore introducing them into the healthcare industry reduces the need for medical professionals while also protecting frontline healthcare workers from coronavirus exposure [30]. The pandemic has increased the number of deaths among healthcare personnel, which has led to a significant rise in the function of receptionists and nursing robots. Three distinct sorts of robots perform the aforementioned roles: medical servers, nurses, and receptionist robots [31].

Recognizing the revolutionary influence of these technologies on healthcare delivery is crucial, particularly about robotic telemedicine and its function in remote healthcare [32]. By bridging the gap between patients and medical experts, robotic devices are enabling people in underserved or rural places to access critical healthcare services. Robots have shown to be highly versatile in the healthcare industry, finding use in telemedicine, social interactions, surgical support, rehabilitation, companionship, and even entertainment. Notably, social robots are made to offer individualized, reasonably priced telemedicine services that support both curative and preventive treatment [33]. The classification of these robots is based on inference-based theories and self-reported qualities, which improves their flexibility in healthcare environments. Healthcare professionals may now give remote patient care to patients from anywhere in the world thanks to telepresence robots, which broadens the scope of medical knowledge. Furthermore, telehealth robots are essential for routinely gathering key data and monitoring patients, which raises the standard and effectiveness of remote medical services. Remote healthcare is being revolutionized by this multifaceted integration of robots, which is advantageous to patients and healthcare providers alike [34].

The Use of Robots in Rehabilitation and Physical Therapy

Rehab robots, also known as assistive and therapeutic robots, are designed to help injured or impaired patients return to their pre-injury state. Helping someone recover from an accident so they can walk again would be a typical scenario [35]. Rehab robots come in a variety of forms and are intended to help patients with a range of conditions. These include stroke, cerebral palsy, and injuries to the knee, ankle, upper and lower limbs, wrist, and elbow [36]. Since studies have demonstrated that children with autism spectrum disorder (ASD) have reacted favorably to therapies including human-robot contact, therapy robots are frequently used in the rehabilitation area [37]. The majority of robots in this category are made with various AI functions that not only treat patients but also keep them motivated. A few of these functions include the ability to play games and recognize facial emotions. These features are designed to make the robots entertaining to both children and the elderly [38].

After an injury or surgery, patients can benefit from the employment of robots to support them with physical rehabilitation activities and help them restore strength and mobility. One tool that assists people with neurological problems or spinal cord injuries in relearning how to walk is the Lokomat robotic gait trainer. The Lokomat can assist patients in achieving better results than would be possible with standard physical therapy alone by offering them regular and repetitive movement patterns [39]. Telerehabilitation has been used more frequently during the COVID-19 pandemic; specifically, rehab robots with cameras and speakers are used for clinical evaluation and monitoring from a distance, further removing the need to endanger the health of patients and physicians [40]. Beyond the previously mentioned features, rehab robots shine at providing individualized rehabilitation plans.

They do this by customizing workouts and resistance levels to meet the specific requirements of each patient, guaranteeing a customized road to recovery. Additionally, by gathering and evaluating performance data to guide treatment decisions and make modifications in real time, these robots maximize the efficacy of therapy. This is known as data-driven rehabilitation. Virtual reality is being used by certain rehab robots to provide engaging and stimulating workouts that improve patient compliance and participation [41]. Additionally, the development of telerehabilitation made possible by robots that have cameras and microphones offers a secure and efficient way to conduct remote clinical evaluation and monitoring. This is especially helpful given the COVID-19 epidemic. Plans call for continued research and development to expand the capabilities of rehab robots, paving the way for a time when these innovations will assist patients on their path to physical rehabilitation with even more accessibility and accuracy [42].

Ethical Considerations in Human-Robot Collaboration

Interaction between people and robots is becoming more frequent in the quickly developing field of robotics. As the contact progresses, privacy, security, and accountability issues are becoming more important [43]. These concerns affect not only the people who utilize these technologies but also the people who create, produce, and oversee them. When it comes to human-robot contact, privacy is crucial. Robots can gather, store, and process enormous volumes of data as they become more advanced. This includes individual people's personal information, which may be utilized in ways that violate their privacy. For example, personal information about a person's eating and sleeping routines may be accessible to home helper robots. If this information ends up in the wrong hands, it could be misused and result in privacy violations [44]. To preserve people's privacy, it is vital to set precise rules for data gathering and use in robots. Another important ethical factor is security. Robots are becoming more and more ingrained in our daily lives, which makes them vulnerable to cyber attacks [45].

Hackers can gain control of a robot and utilize it for malicious purposes, such as bodily injury or data theft. This begs the question of how to safeguard people from possible danger and guarantee the security of robots. Strong security features must be incorporated into designs as a top priority for manufacturers, and they must be updated often to fend off new threats. The topic of accountability in the context of human-robot interaction is complicated. Who is at fault if a robot hurts someone? Who is to blame the maker, the coder, the end user, or the robot itself? The response is not simple to understand [46]. It could be a mix of these elements in many circumstances. Yet it gets harder and harder to assign blame to robots as they become more independent. This is especially relevant in the context of artificial intelligence, where the decisions made by a robot could be the product of machine learning algorithms that not even its designers completely comprehend. The ethical issues surrounding human-robot contact are intricate and varied. They necessitate a multidisciplinary strategy comprising ethicists, attorneys, and policymakers in addition to engineers. Furthermore, they necessitate constant discussion and debate since new ethical conundrums arise and technology keeps developing [47].

Challengers and Barriers to Successful Implementation

Human-robot collaboration for healthcare faces several obstacles and issues that must be carefully considered to be implemented successfully. The ethical and legal environment around the use of robots in healthcare is one of the main obstacles. To guarantee the safe and responsible implementation of new technologies, concerns about patient privacy, data security, informed permission, and responsibility must be thoroughly addressed [48]. Furthermore, many healthcare facilities, especially smaller, underfunded ones, may find it difficult to afford the upfront costs associated with obtaining and integrating robotic equipment, let alone the continuing maintenance and training requirements. For various robotic platforms and medical equipment to work together seamlessly, interoperability and standardization of robotic systems and interfaces are essential. Another crucial factor to take into account is making sure that robots enhance, not replace, the knowledge and experience of healthcare workers. To overcome these obstacles, a multidisciplinary team comprising physicians, engineers, legislators, and ethicists must develop policies, guidelines, and best practices that support the moral and practical integration of robots into healthcare, ultimately improving patient care and well-being [49].

As a result, there is a lot of exciting potential and innovation for human-robot collaboration in healthcare in the future. We must continue to be watchful in addressing the ethical, legal, and practical concerns that lie ahead as we investigate and utilize robotics in healthcare. We can fully utilize these technologies by collaborating across disciplines, with the ultimate goal of improving patient care, expanding access to healthcare, and offering more individualized and efficient medical solutions to everyone. A summary of all articles included in this review is listed in Table 1.

**Table 1:** Summary table of studies included in the review

Authors	Year	Country	Findings
Holland J et al. [2]	2021	Ireland	The use of assistive technology in healthcare is on the rise, offering the potential to save time and enhance accuracy in medical procedures.
Gleichauf K et al. [3]	2022	Germany	Anticipated growth in human-robot collaboration in healthcare aims to address the shortage of medical personnel due to the aging population, with a focus on promoting technology acceptance among the medical team.
Bedaf S et al. [4]	2017	Netherlands	CoBots assist in rehabilitation, mental health support, and aiding individuals with impairments in healthcare settings.
Shen Y et al. [5]	2020	USA	CoBots address healthcare personnel burnout and enhance telehealth, especially during the COVID-19 pandemic, by facilitating safe human-robot interactions.
Arif D et al. [6]	2017	Pakistan	Telehealth robots improve communication between patients and healthcare professionals, record vital data, monitor patient conditions, and provide emergency alerts, enhancing patient care.
Su H et al. [7]	2021	USA	Robots collaborate with doctors and patients, acting as therapists when necessary, and trust in human-robot interaction is a growing topic within the medical community, ensuring optimal healthcare outcomes.
González-González CS et al. [8]	2021	Spain	Assistive robots are mostly prototypes, but ongoing research aims to create versatile interactive companions with various capabilities.
Javaid M et al. [9]	2022	India	Collaborative robots show great promise in the medical field, particularly in automating routine and low-value tasks performed by healthcare professionals.
Raza MH et al. [11]	2022	Pakistan	The evolution of robots in the field of pharmacy offers the potential to streamline operations by simplifying robot interfaces and enabling task customization. As technology advances, robots are poised to revolutionize pharmacy practices, enhancing patient safety and efficiency.
D’Onofrio G et al. [13]	2023	Italy	Medical robotics offers three key benefits: reducing contact with infected patients to prevent disease spread, increasing clinical efficiency, and minimizing the need for personal protective equipment. The current state of medical robotics plays a pivotal role in addressing these healthcare concerns.
Soriano GP et al. [14]	2022	Japan	Advanced technologies to create adaptable robots for future infectious disease outbreaks. Improvements in autonomy, manipulation, and sensing are vital for enhancing the robots' capabilities
Morris B et al. [15]	2005	Egypt	Surgical robots are computer-controlled devices that assist surgeons by handling surgical tools and facilitating complex tasks during surgery.
Lanfranco AR et al. [16]	2004	USA	Robotic surgery, exemplified by the da Vinci Surgical System, enhances surgical precision and patient outcomes through improved surgeon control and dexterity.
Khajuria A et al. [17]	2015	United Kingdom	Robotic surgical systems enable minimally invasive surgeries, reducing patient recovery times, blood loss, and hospital stays. They also contribute to enhanced patient outcomes by minimizing the chances of human error and surgeon fatigue.
Shah J et al. [18]	2015	USA	Robotic assistance in medicine, including surgery, is a groundbreaking development across medical specialties, enhancing surgical capabilities and benefiting patient outcomes.
Thimbleby et al. [19]	2013	United Kingdom	Financial challenges faced by healthcare facilities in procuring and maintaining robotic surgical equipment, emphasizing the importance of enhancing accessibility measures for robotic technologies.
Dobra Z et al. [20]	2023	Germany	Role of advanced technologies like augmented reality, haptic feedback, and AI-driven decision support in enhancing healthcare practices.
Wang Z et al. [21]	2023	Cyprus	Legal considerations that come with the technological revolution in healthcare involving robots.
Naceri A et al. [22]	2022	Germany	Telemedicine in providing healthcare services to residents in rural and isolated areas.
Townsend D et al. [28]	2022	USA	Various applications of robots in the healthcare sector, including telemedicine, social contact, surgical support, rehabilitation, companionship, and entertainment.
Allan DD et al. [26]	2022	Zealand	Use of social robots designed to provide personalized, cost-effective telemedicine services for both curative and preventive care.
Wong L et al. [27]	2023	USA	Telepresence robots in the healthcare sector, which enable medical practitioners to provide patient care from remote locations.
Esterwood C et al. [24]	2023	USA	Telehealth robots can be used to provide patient monitoring and management such as daily vital measurements
Deo N et al. [25]	2023	India	Robots in healthcare assist with supply transport and cleaning duties, improving efficiency and hygiene in hospitals
Raje S et al. [30]	2021	India	Robots in healthcare can reduce the need for medical professionals and protect healthcare workers from COVID-19 exposure.
Sodhi GK et al. [31]	2022	India	Due to the pandemic, the demand for receptionists and nursing robots has increased, with roles performed by medical servers, nurses, and receptionist robots
Haleem A et al. [32]	2021	India	Recognizing the transformative impact of these technologies on healthcare delivery, particularly in the context of robotic telemedicine and its role in remote healthcare, is essential.
Thinh NT et al. [33]	2021	Viet Nam	Robots are versatile in healthcare, serving in telemedicine, social interactions, surgery support, rehabilitation, companionship, and entertainment. Social robots focus on personalized, affordable telemedicine for both curative and preventive healthcare.
Terashima K et al. [34]	2022	Japan	The multifaceted integration of robots is revolutionizing remote healthcare, benefiting both patients and healthcare providers
Polak RF et al. [35]	2020	Israel	Rehab robots, also known as assistive and therapeutic robots, aim to assist injured or impaired patients in their recovery, often helping them regain the ability to walk.
Buxbaum H et al. [36]	2019	Germany	Rehab robots come in a variety of forms and are intended to help patients with a range of conditions.
Coeckelbergh M et al. [37]	2015	UK	Rehabilitation robots come in various forms and are designed to aid patients with a wide range of conditions, including stroke, cerebral palsy, and injuries affecting the knee, ankle, upper and lower limbs, wrist, and elbow.
Tanguay P et al. [38]	2021	Canada	Most robots in the rehabilitation category incorporate various artificial intelligence (AI) functions that not only assist in patient treatment but also help keep patients motivated. These functions may include playing games and recognizing facial emotions, enhancing the robots' appeal to both children and the elderly.
Nam KY et al. [39]	2017	Korea	The Lokomat is capable of helping patients achieve improved outcomes compared to standard physical therapy alone, primarily by providing them with regular and repetitive movement patterns.
Isabet B et al. [40]	2021	France	The COVID-19 pandemic has led to increased use of telerehabilitation, including rehab robots with cameras and speakers for remote clinical evaluation and monitoring, ensuring safety for patients and physicians.
Hatem SM et al. [41]	2016	Belgium	Some rehab robots use virtual reality to deliver engaging and stimulating workouts, enhancing patient compliance and participation in the rehabilitation process.
Wang R et al. [42]	2023	China	Telerehabilitation, facilitated by robots equipped with cameras and microphones, provides a secure and efficient method for conducting remote clinical evaluation and monitoring, a particularly valuable approach during the COVID-19 pandemic
Boada JP et al. [43]	2021	Spain	With the growing interaction between people and robots in the rapidly advancing field of robotics, issues related to privacy, security, and accountability are gaining increasing significance
Fosch-Villaronga E et al. [44]	2021	Netherlands	As robots become more advanced, they can collect extensive personal data, including sensitive information like eating and sleeping habits. This raises concerns about potential privacy violations and misuse if this data is mishandled
Etemad-Sajadi R et al. [45]	2021	Switzerland	Preserving privacy requires clear rules for data collection and use in robots, while cybersecurity is crucial as robots become more integrated into daily life, making them susceptible to cyberattacks
Shahid J et al. [46]	2022	Pakistan	Hackers can take control of robots for malicious purposes, posing risks of physical harm and data theft.
Cresswell K et al. [48]	2018	United Kingdom	Ethical and legal issues, including patient privacy and data security, pose significant challenges to the successful implementation of Human-Robot Collaboration for Healthcare and must be carefully addressed for responsible use.
Lawrie L et al. [49]	2022	United Kingdom	Healthcare facilities, especially smaller and underfunded ones, face financial challenges in acquiring, integrating, and maintaining robotic equipment due to high initial costs and ongoing training requirements.
Borboni A et al. [43]	2023	Italy	The ethical issues surrounding human-robot contact are intricate and varied
Wallace J [1]	2021	Denmark	Cobots, or collaborative robots, emphasise that its goal is to securely work with humans in a shared office.
Javaid M et al. [10]	2022	India	Cobots help with pick-and-place jobs in manufacturing, support medical duties, help with warehouse operations, and find use in agriculture. Their potential to improve human capacities and establish safer, more productive work conditions is being explored further.
Mehta A et al. [23]	2022	Hungary	Prospective studies show that robotically assisted surgery can have positive results, particularly when using devices such as the da Vinci Surgical System. Less blood loss, fewer incisions, and shorter hospital stays are possible outcomes for patients undergoing such treatments. Its effectiveness is not absolute, though; the intricacy of the surgery and the surgeon's skill level are important considerations.

## Conclusions

Human-robot collaboration for healthcare, to put it briefly, signifies a revolutionary change in the way healthcare is provided. Robotics' growing incorporation into the healthcare system presents opportunities to improve patient care, maximize productivity, and tackle the most important issues facing the field today. The future of seamless human-robot collaboration promises more accessible and patient-centered healthcare, despite practical, ethical, and legal challenges. We can realize the full potential of robotics in healthcare by overcoming these obstacles and promoting interdisciplinary collaboration, which will eventually improve the health of patients and healthcare workers alike.

## References

[1] Wallace J (2021). Getting collaborative robots to work: a study of ethics emerging during the implementation of cobots. Paladyn J Behav Robot.

[2] Holland J, Kingston L, McCarthy C, Armstrong E, O’Dwyer P, Merz F, McConnell M (2021). Service robots in the healthcare sector. Robotics.

[3] Hartl VW, Gleichauf K, Schmid R, Dewald N (2023). The impact of the COVID-19 pandemic on attitudes towards human-robot collaboration in the healthcare environment. AHFE International.

[4] Bedaf S, Marti P, De Witte L (2019). What are the preferred characteristics of a service robot for the elderly? A multi-country focus group study with older adults and caregivers. Assist Technol.

[5] Shen Y, Guo D, Long F (2020). Robots under COVID-19 pandemic: a comprehensive survey. IEEE Access.

[6] Arif D, Ahmad A, Bakar M, Ihtisham M, Winberg S (2017). Cost effective solution for minimization of medical errors and acquisition of vitals by using autonomous nursing robot. Proc Int Conf Inf Sys Data Min.

[7] Sefati S, Hegeman R, Iordachita I, Taylor RH, Armand M (2022). A dexterous robotic system for autonomous debridement of osteolytic bone lesions in confined spaces: human cadaver studies. IEEE Trans Robot.

[8] González-González CS, Violant-Holz V, Gil-Iranzo RM (2021). Social robots in hospitals: a systematic review. Appl Sci.

[9] Javaid M, Haleem A, Singh RP, Rab S, Suman R (2022). Significant applications of cobots in the field of manufacturing. Cogn Robot.

[10] Javaid M, Haleem A, Singh RP, Suman R (2021). Significant applications of cobots in the field of manufacturing. Cogn Robot.

[11] Raza MA, Aziz S, Noreen M, Saeed A, Anjum I, Ahmed M, Raza SM (2022). Artificial intelligence (AI) in pharmacy: an overview of innovations. Innov Pharm.

[12] (2018). Cobots in the pharmaceutical industry. https://www.wiredworkers.io/cobot/industries/pharmaceutical-industry.

[13] D'Onofrio G, Sancarlo D (2023). Assistive robots for healthcare and human-robot interaction. Sensors (Basel).

[14] Soriano GP, Yasuhara Y, Ito H (2022). Robots and robotics in nursing. Healthcare (Basel).

[15] Morris B (2005). Robotic surgery: applications, limitations, and impact on surgical education. MedGenMed.

[16] Lanfranco AR, Castellanos AE, Desai JP, Meyers WC (2004). Robotic surgery: a current perspective. Ann Surg.

[17] Khajuria A (2015). Robotics and surgery: a sustainable relationship?. World J Clin Cases.

[18] (2004). Computer-assisted surgery using telemanipulators: an evidence-based analysis. Ont Health Technol Assess Ser.

[19] Shah J, Vyas A, Vyas D (2014). The history of robotics in surgical specialties. Am J Robot Surg.

[20] Thimbleby H (2013). Technology and the future of healthcare. J Public Health Res.

[21] Dobra Z, Dhir K (2020). Technology jump in the industry: human-robot cooperation in production. Ind Robot Int J Robot Res Appl.

[22] Stoumpos AI, Kitsios F, Talias MA (2023). Digital transformation in healthcare: technology acceptance and its applications. Int J Environ Res Public Health.

[23] Mehta A, Cheng Ng J, Andrew Awuah W (2022). Embracing robotic surgery in low- and middle-income countries: potential benefits, challenges, and scope in the future. Ann Med Surg (Lond).

[24] Naceri A, Elsner J, Trobinger M (2022). Tactile robotic telemedicine for safe remote diagnostics in times of corona: system design, feasibility and usability study. IEEE Robot Autom Lett.

[25] Olaronke I, Ojerinde OA, Ikono R (2023). State of the art: a study of human-robot interaction in healthcare. Int J Inf Eng Electron Bus.

[26] Allan DD, Vonasch A, Bartneck C (2022). The doors of social robot perception: the influence of implicit self-theories. Int J Soc Robot.

[27] Wong L, Tokumaru S, Boehm L (2021). From a distance: nursing and pharmacy students use teamwork and telehealth. J Interprofessional Educ Pract.

[28] Esterwood C, Robert LP (2023). The theory of mind and human-robot trust repair. Sci Rep.

[29] Deo N, Anjankar A (2023). Artificial intelligence with robotics in healthcare: a narrative review of its viability in India. Cureus.

[30] Raje S, Reddy N, Jerbi H (2021). Applications of healthcare robots in combating the COVID-19 pandemic. Appl Bionics Biomech.

[31] Sodhi GK, Kaur S, Gaba GS, Kansal L, Sharma A, Dhiman G (2022). COVID-19: role of robotics, artificial intelligence and machine learning during the pandemic. Curr Med Imaging.

[32] Haleem A, Javaid M, Singh RP, Suman R (2021). Telemedicine for healthcare: capabilities, features, barriers, and applications. Sens Int.

[33] Thinh NT, Hai ND (2021). Telemedicine mobile robot - robots to assist in remote medical. Int J Mech Eng Robot Res.

[34] Terashima K, Funato K, Komoda T (2022). Healthcare Robots and Smart Hospital Based on Human-Robot Interaction. J hum robot interact.

[35] Feingold Polak R, Levy-Tzedek S (2020). Social robot for rehabilitation: expert clinicians and post-stroke patients’ evaluation following a long-term intervention. ACM/IEEE Conference Human-Robot Interaction.

[36] Buxbaum H, Sen S, Kremer L (2019). An investigation into the implication of human-robot collaboration in the health care sector. IFAC-Pap.

[37] Coeckelbergh M, Pop C, Simut R, Peca A, Pintea S, David D, Vanderborght B (2016). A survey of expectations about the role of robots in robot-assisted therapy for children with ASD: ethical acceptability, trust, sociability, appearance, and attachment. Sci Eng Ethics.

[38] Tanguay P, Marquis N, Gaboury I, Kairy D, Touchette M, Tousignant M, Décary S (2021). Telerehabilitation for post-hospitalized COVID-19 patients: a proof-of-concept study during a pandemic. Int J Telerehabil.

[39] Nam KY, Kim HJ, Kwon BS, Park JW, Lee HJ, Yoo A (2017). Robot-assisted gait training (Lokomat) improves walking function and activity in people with spinal cord injury: a systematic review. J Neuroeng Rehabil.

[40] Isabet B, Pino M, Lewis M, Benveniste S, Rigaud AS (2021). Social telepresence robots: a narrative review of experiments involving older adults before and during the COVID-19 pandemic. Int J Environ Res Public Health.

[41] Hatem SM, Saussez G, della Faille M, Prist V, Zhang X, Dispa D, Bleyenheuft Y (2016). Rehabilitation of motor function after stroke: a multiple systematic review focused on techniques to stimulate upper extremity recovery. Front Hum Neurosci.

[42] Wang R, Lv H, Lu Z, Huang X, Wu H, Xiong J, Yang G (2023). A medical assistive robot for telehealth care during the COVID-19 pandemic: development and usability study in an isolation ward. JMIR Hum Factors.

[43] Boada JP, Maestre BR, Genis CT (2023). The ethical issues of social assistive robotics: a critical literature review. Technol Soc.

[44] Eduard FV, Tobias M (2021). Cybersecurity, safety and robots: strengthening the link between cybersecurity and safety in the context of care robots. Comput Law Secur Rev.

[45] Etemad-Sajadi R, Soussan A, Schöpfer T (2022). How ethical issues raised by human-robot interaction can impact the intention to use the robot?. Int J Soc Robot.

[46] Shahid J, Ahmad R, Kiani AK, Ahmad T, Saeed S, Almuhaideb AM (2022). Data protection and privacy of the internet of healthcare things. Appl Sci.

[47] Borboni A, Reddy KV, Elamvazuthi I, Quraishi AL, Natarajan E, Ali SS (2023). The expanding role of artificial intelligence in collaborative robots for industrial applications: a systematic review of recent works. Machines.

[48] Cresswell K, Cunningham-Burley S, Sheikh A (2018). Health care robotics: qualitative exploration of key challenges and future directions. J Med Internet Res.

[49] Lawrie L, Gillies K, Duncan E, Davies L, Beard D, Campbell MK (2022). Barriers and enablers to the effective implementation of robotic assisted surgery. PLoS One.

